# Unpacking non-transparency: Most physical trading card game and collectible sports card packs failed to disclose probabilities for winning rare prizes

**DOI:** 10.1556/2006.2026.00197

**Published:** 2026-08-18

**Authors:** Ching Yiu Lo, Kuma Xiaoyu Xiong, Leon Y. Xiao

**Affiliations:** 1 School of Creative Media, City University of Hong Kong, China; 2 BeClaws.org, UK

**Keywords:** card packs, blind boxes, physical gambling-like products, information disclosure, risk warning and communication

## Abstract

**Background and aims:**

Physical card packs are gambling-like products offering consumers random prizes. Most contain cards of little value worth less than the purchase price of the pack itself, but a few contain rare and highly valuable cards. Previous research has found a weak positive association between physical card pack spending and problem gambling, suggesting that vulnerable consumers may experience financial harm and that these physical gambling-like products may pose addiction-like risks. Unlike traditional gambling, card packs are not an adult-only product and are widely purchased by children too. Worldwide, there is currently no dedicated card pack regulation. Notwithstanding, basic consumer law applies, requiring companies to disclose and not hide important information about the product: specifically, the odds of obtaining different prizes.

**Methods:**

We sampled 50 card packs in Hong Kong representing popular products consumers buy to assess whether they disclosed winning probabilities.

**Results:**

The vast majority (80.0%) failed to provide any probability disclosure information or relevant hyperlinks on their physical packaging. The other 20.0% that provided probability disclosures failed to give sufficient details and were, occasionally, difficult to find and misleading. No pack (0.0%) allowed the consumer to know their odds of getting every individual card.

**Discussion and conclusions:**

In the absence of gambling law and dedicated regulations, consumer law should be better enforced against gambling-like products (including physical Pokémon card packs, Labubu blind boxes, and video game loot boxes) to mandate transparency and enable consumers to make more informed purchasing decisions. The potential harms posed by novel gambling-like products should be further studied and properly regulated.

## Introduction

Card packs are physical gambling-like products that consumers purchase to obtain a random selection of collectible cards of varying rarity and value (Zendle, Walasek, Cairns, Meyer, & Drummond, 2021). Rarer cards often are worth more money on secondary markets, *e.g.*, on eBay (Heck et al., 2026; Ward & Clark, 2002), where cards are traded between players after having initially been obtained through pack openings (Hughes, 2022). Some card packs, such as for sports, are purely for collecting and do not offer any accompanying gameplay experiences (Parrott, 2026), whilst other card packs provide crucial gameplay pieces for a genre of physical tabletop games known as collectible or trading card games (CCGs or TCGs): for example, *Magic: The Gathering* (Wizards of the Coast, 1993–Present) and the *Pokémon Trading Card Game* (The Pokémon Company, 1996–Present) (Calvo, Carbonell, Oberst, & Fuster, 2018; Mattinen, Macey, & Hamari, 2023). Physical card packs are internationally popular, having experienced a resurgence during and following the Covid-19 pandemic (Ang, 2025; Igarashi, 2026; Lucas, 2025), and constitute a multi-billion-dollar global industry (Muroi, 2026; Xiao, Zendle, Petrovskaya, Nielsen, & Newall, 2026, pp. 68–69).

Because the value of potential cards obtainable from card packs differ greatly, with most packs containing cards whose total value is less than the purchase price of the sealed pack but a small minority of packs containing highly valuable cards (Xiao et al., 2026, pp. 67–68), concerns have been raised as to whether these products are structurally and psychologically similar to gambling (Zendle et al., 2021). Reddit users with lived experience as consumers have commented on the ‘addictive’ nature of physical card packs and described observing others experiencing financial and social harm as a result of their engagement (Parrott, 2026, p. 347). These products may indeed already fall under existing legal definitions of ‘gambling’ across jurisdictions because they involve the consumer staking money on a random outcome offering prizes that can be converted into real-world money, although the law is not enforced in practice (Elliott & Mason, 2003; Xiao, 2022). Consequently, these products remain widely available for purchase, including by young children and their parents, without legally-mandated age restrictions, unlike traditional gambling.

Limited psychology research has been conducted (Sanders, Stevens, Radünz, Delfabbro, & King, 2026, p. 9), suggesting that spending on physical card packs is weakly correlated with problem gambling at a level that, whilst arguably practically significant, would not be highly concerning, as compared to digital gambling-like products, such as video game ‘loot boxes’ that offer random virtual prizes (Spicer et al., 2022), which appeared more harmful (Xiao et al., 2026; Zendle et al., 2021). Indeed, spending money on physical card packs was not associated with negative mental health (Xiao et al., 2026), whilst spending money on digital loot boxes is associated with experiencing greater psychological distress, depending on how the data are analysed (Drummond, Hall, Lowe-Calverley, Garrett, & Sauer, 2025; cf. Etchells, Morgan, & Quintana, 2022; Xiao, Fraser, Nielsen, & Newall, 2024).

Importantly, consumers are motivated to spend money on physical card packs for a variety of different reasons, including not just to obtain expensive cards to resell for profit, but also growing their collection by adding new cards that unlock novel gameplay experiences without being particularly concerned about monetary gains or losses; playing in tournaments requiring packs to be opened and only cards from those packs to be used in so-called ‘booster drafts’ (Wizards of the Coast, 2026) and ‘sealed’ formats (Wizards of the Coast, 2023a); or even gifting packs to their family and friends without engaging with gambling-like aspects of the product at all. Undoubtedly, these latter motivations are less similar to traditional gambling and less obviously harmful than attempting to open packs exclusively for monetary gain. This diverse range of different engagement experiences may explain the less pronounced correlation and should be further explored.

Irrespective of potential psychological harms, consumer law applies to physical card packs as a commercial product sold to the general public. One important aspect is that consumers must be duly informed of any material information about the product that they need to make an informed purchase decision. In the videogaming loot box context, many jurisdictions, including Mainland China (Xiao, Henderson, Yang, & Newall, 2024), South Korea (Xiao & Park, 2025), the UK (Xiao, Henderson, & Newall, 2023; Xiao & Lund, 2025), and the EU (Xiao, 2025), now require companies to inform players of their exact probabilities of obtaining various potential rewards, so that players can perhaps appreciate how unlikely they are to obtain the rare and valuable prizes and how likely they are to instead ‘lose’ money by purchasing these products and only obtaining undesirable prizes.

In particular, the European Commission (2021, p. 105) has explicitly and authoritatively interpreted EU consumer law (Article 7 of the Unfair Commercial Practices Directive 2005) to require loot box probability disclosures, as the probabilities of winning different prizes are viewed as ‘material information’ whose omission is impermissible. For historical reasons, particularly in relation to so-called ‘misleading omissions’ (*i.e.*, failing to provide or hiding material information), near identical consumer laws were transposed and currently apply in other jurisdictions, such as the UK (Section 227 of the Digital Markets, Competition and Consumers Act 2024) and Hong Kong (Section 13E of the Trade Descriptions Ordinance) (Durovic, Howells, & Janssen, 2019, p. 594). Similar consumer law provisions exist in many other jurisdictions, like the US (Section 5(a) of the Federal Trade Commission Act) (Federal Trade Commission (US), 2025). By the same logic, the probabilities associated with winning rare prizes from physical card packs are also material information whose disclosure is required under consumer law. The Hong Kong Government has confirmed that the Trade Descriptions Ordinance, including specifically the prohibition on misleading omissions, applies to physical ‘blind boxes’ offering random toys like Labubus (Yau, 2025), confirming that our reasoning by analogy is sound.

Whilst a wealth of research has been conducted on virtual gambling-like products, including specifically in relation to probability disclosure provision in various jurisdictions (Xiao, 2025; Xiao et al., 2023; Xiao, Henderson, et al., 2024; Xiao & Lund, 2025; Xiao & Park, 2025), no prior research has systematically examined the product characteristics of gambling-like physical card packs and their compliance with transparency requirements under consumer law, *i.e.*, whether winning probabilities are disclosed. We address this research gap by scrutinising this multi-billion-dollar, potentially behaviourally addictive industry that has been subject to little academic inquiry (Sanders et al., 2026, p. 9).

## Method

In October 2025, we purchased 50 different physical card packs in Hong Kong. Because randomised sampling was not practicable as thousands of different products are for sale, our sampling was mostly based on convenience: we bought products that were easily obtainable from physical shops. We also sampled purposively by choosing to purchase certain products that are known to be widely popular based on prior research that surveyed players about the TCGs whose card packs they spent money on (*e.g.*, *Pokémon* and *Magic: The Gathering*) (Xiao et al., 2026, p. 78). Simultaneously, we also tried to purchase certain products that are as different as possible when compared to others to gather a more diverse sample.

We then examined the physical products' packaging to search for any information regarding the probabilities of obtaining specific rewards from the card pack, including potentially a hyperlink to probability disclosures on a webpage. We also separately searched for official probability disclosure information online using Google.

Inter-rater reliability was very high as to whether probability disclosures (Cohen's *κ* = 1.00) and a hyperlink to probability disclosures on a webpage (Cohen's *κ* = 0.97) were present on the physical packaging. The single disagreement was revealed to be a false negative following discussion, and both coders agreed on the final coding and an amendment to the codebook to eliminate any confusion during future use.

We preregistered this study at https://doi.org/10.17605/OSF.IO/97ZNM and publicly shared our data via https://doi.org/10.17605/OSF.IO/ZP7SX.

### Ethics

This study did not require research ethics assessment and approval because no human participants or personal data were expected to have been involved and only publicly available information was examined and recorded.

## Results

### Sample characteristics

Our sample consisted of nearly all recent products, 92.0% of which were released within three years of the data collection period. Most (72.0%) card packs we studied related to a Japanese intellectual property (IP), although other regions' IP were also present with the US having a significant minority (18.0%). The packs were also mostly physically manufactured in Japan (74.0%). Most (64.0%) packs contained Japanese cards, whilst a minority offered English (24.0%) and Chinese (12.0%) cards. Most (76.0%) card packs showed an age rating and were deemed suitable for children (*M* = 10.89, *SD* = 3.17). The cards from 40 packs (80.0%) were designed to be played as part of a game: 39 packs were for TCGs, whilst one pack was for an arcade game. The cards from the other 10 packs (20.0%) were exclusively designed for collecting only, such as sports cards. We paid a small amount ranging between HK$5–100 (≈ US$0.65–12.75) each for these packs (*M* = 33.90, *SD* = 18.46), accurately reflecting the usual prices for such products. Table 1 details the sample characteristics. Considering the above, our sample of recently released physical card packs is representative of the usual products consumers purchase in Hong Kong, where Japanese products are generally more popular, but a limited selection of English and Chinese products is also sold.

**Table 1.** Card pack sample characteristics (*N* = 50)

Characteristic	# (%)
**Release year**	
2025	29 (58.0%)
2024	14 (28.0%)
2023	3 (6.0%)
2022	1 (2.0%)
2021	1 (2.0%)
2013	1 (2.0%)
2004	1 (2.0%)
**Origin of the intellectual property**	
Japan	36 (72.0%)
US	8 (16.0%)
US/UK (crossover product)	1 (2.0%)
Canada	1 (2.0%)
New Zealand	1 (2.0%)
China	1 (2.0%)
International	2 (4.0%)
**Physically manufactured in**	
Japan	37 (74.0%)
US	4 (8.0%)
China	3 (6.0%)
Singapore	1 (2.0%)
Belgium	1 (2.0%)
Italy	1 (2.0%)
Not provided	3 (6.0%)
**Language**	
Japanese	32 (64.0%)
English	12 (24.0%)
Traditional Chinese	3 (6.0%)
Simplified Chinese	3 (6.0%)
**Age rating**	
3	1 (2.0%)
6	2 (4.0%)
8	5 (10.0%)
9	10 (20.0%)
10	2 (4.0%)
12	2 (4.0%)
13	8 (16.0%)
15	7 (14.0%)
16	1 (2.0%)
Not provided	12 (24.0%)
**Designed for gameplay?**	
No (Collectible only)	10 (20.0%)
Sports cards	5 (10.0%)
Film or television programmes (*e.g.*, anime)	5 (10.0%)
Yes (Arcade Game)	1 (2.0%)
*Super Dragon Ball Heroes*	1 (2.0%)
Yes (Tabletop game)	39 (78.0%)
*Magic: The Gathering*	4 (8.0%)
*Pokémon Trading Card Game*	4 (8.0%)
*Angelic Link Trading Card Game*	1 (2.0%)
*Animal Card Game*	1 (2.0%)
*Battle Spirits*	1 (2.0%)
*Build Divide*	1 (2.0%)
*Carddass Chiikawa*	1 (2.0%)
*Detective Conan Card Game*	1 (2.0%)
*Digimon Card Game*	1 (2.0%)
*Disney Lorcana*	1 (2.0%)
*Dragon Ball Super Card Game Fusion World*	1 (2.0%)
*Duel Masters*	1 (2.0%)
*Final Fantasy Trading Card Game*	1 (2.0%)
*Flesh and Blood*	1 (2.0%)
*Force of the Horse*	1 (2.0%)
*Godzilla Card Game*	1 (2.0%)
*Gotoubun no Hanayome Card Game*	1 (2.0%)
*Gundam Card Game*	1 (2.0%)
*Gundam War*	1 (2.0%)
*Hololive Official Card Game*	1 (2.0%)
*Match Attax*	1 (2.0%)
*OSICA*	1 (2.0%)
*ONE PIECE Card Game*	1 (2.0%)
*Shadowverse: Evolve*	1 (2.0%)
*SiegKrone*	1 (2.0%)
*Star Wars: Unlimited*	1 (2.0%)
*Trails Trading Card Game*	1 (2.0%)
*Union Arena*	1 (2.0%)
*WIXOSS*	1 (2.0%)
*Weiß Schwarz*	1 (2.0%)
*Xross Stars*	1 (2.0%)
*Yu-Gi-Oh!*	1 (2.0%)
*Z/X -Zillions of enemy X-*	1 (2.0%)

### Probability disclosure compliance

Amongst the 50 card packs, only 15 packs (30.0%) ostensibly provided some information relating to the probabilities of obtaining different cards: seven packs (14.0%) had probability disclosures printed on their physical packaging; four packs (8.0%) ostensibly provided a hyperlink that is supposed to lead to the probability disclosures on a webpage; and four packs (8.0%) both had probabilities printed on them and ostensibly provided a hyperlink to webpage-based probability disclosures. However, when trying to use the eight hyperlinks provided to find the relevant probability disclosure information, we were only eventually successful in three cases. All three cases required significant efforts to navigate the relevant website, as none of the hyperlinks provided were direct links: we had to follow multiple links and review various webpages, and it was often unclear which link needed to be followed and where the correct link would be located on a certain webpage. For the other five cases, we were unable to find relevant probability disclosures by following the link and navigating through the website despite reasonable efforts.

Consequently, only 10 packs (20.0%) actually provided any probability disclosures. Upon closer inspection, however, these disclosures were not sufficiently detailed, as shown in Fig. 1. For example, some packs only provided the probabilities for rare and highly sought-after ‘chase’ cards and failed to disclose the probabilities for obtaining less rare cards. Whilst the former is admittedly likely more important for consumers to know, the latter is also relevant information that consumers should be given. Further, some packs potentially offered cards of multiple rarities, but instead of providing separate probabilities for each rarity, only provided one combined probability for multiple rarities, making it impossible for the consumer to know their exact probabilities for obtaining cards of a specific rarity. We also observed card packs disclosing inexact and arguably misleading probabilities, such as ‘<1%,’ which potentially disguises an extremely low probability like ‘0.00012492%’ as being ‘just under 1%,’ as has also been observed in video game loot box contexts (see Xiao, 2025, p. 20).

**Fig. 1.**
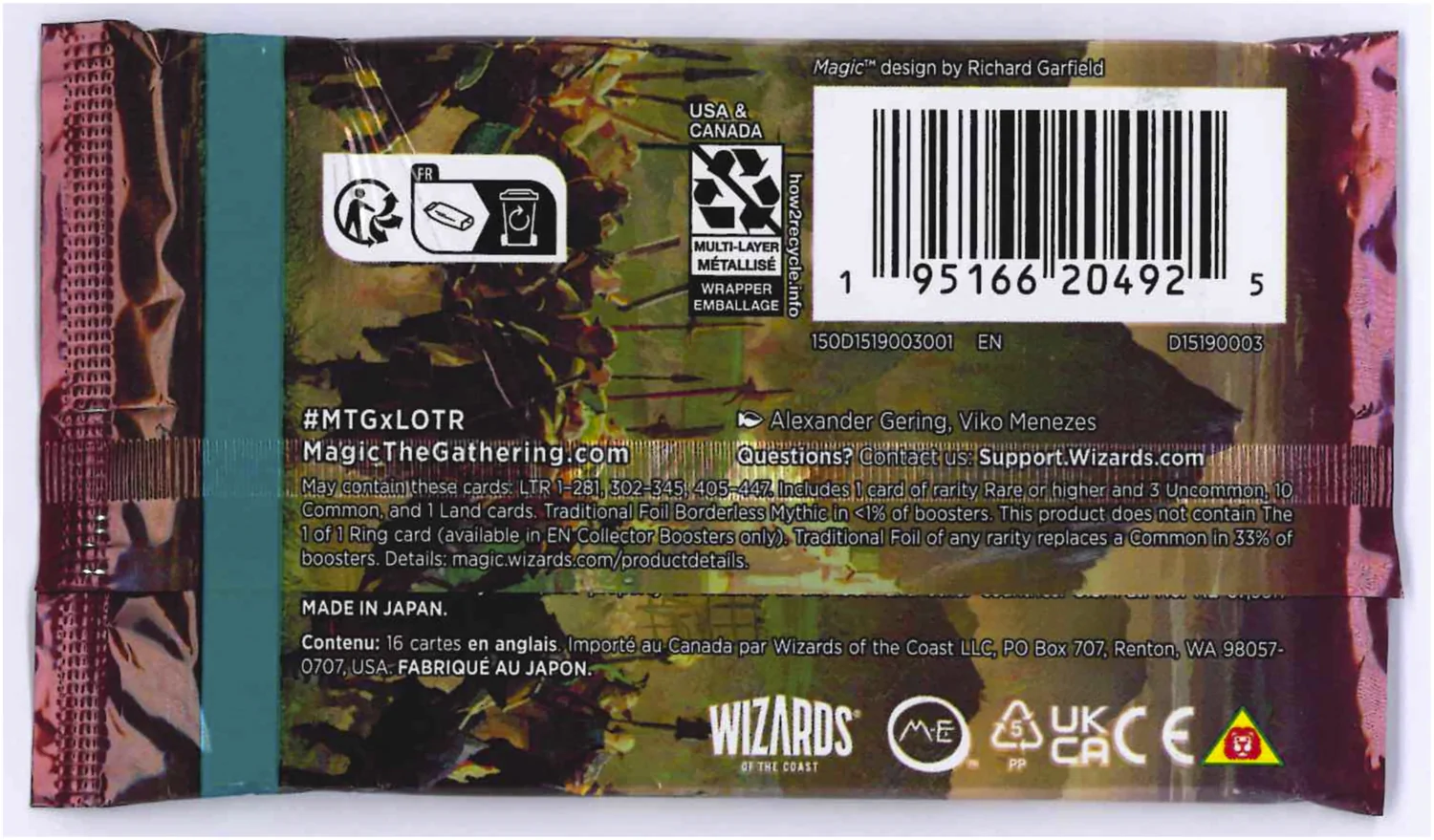
A *Magic: The Gathering* crossover *The Lord of the Rings: Tales of Middle-earth* Draft Booster Pack disclosed limited probability-related information on its physical packaging. However, the disclosure was not sufficiently detailed, failing to provide exact probabilities. The associated online webpage, which was not directly linked to and was difficult to find, also failed to provide sufficient details (Wizards of the Coast, 2023c). © 2023 Wizards of the Coast & Middle-earth Enterprises

Through Google searches, we additionally found probability disclosures for three other packs (6.0%). However, these websites disclosures were similarly inadequate by failing to provide sufficient details as described above. Importantly, the links to the relevant webpages were not provided on the physical card pack packaging and thus were not provided to consumers sufficiently timely or prominently prior to purchase. By requiring consumers to separately search for this important information at their own volition, rather than providing it by default, the relevant manufacturers likely breached consumer law.

No card pack (0.0%) provided (on its physical packaging or, acknowledging the limited space available there, associated webpages whose links were printed on the packaging) detailed disclosures as to the probabilities for obtaining each individual possible card. If a consumer wants to know their probabilities of obtaining every individual card, they simply cannot. Therefore, it was also impossible for the consumer to estimate how many packs they need to purchase and how much money they need spend on average to obtain a specific desired card, thus hindering them from making informed purchase decisions.

## Discussion

Physical card packs are gambling-like products that offer random cards in exchange for money, with the consumer far more often obtaining cards that are worth less than the purchase price of the pack. Similar gambling-like products in virtual contexts, such as video game loot boxes and gacha mechanics, have recently been subject to significant controversy and academic and regulatory scrutiny (Leahy, 2022), leading to many countries now requiring companies to disclose the probabilities for obtaining different prizes (Xiao, Henderson, Nielsen, & Newall, 2022). Whilst policy implementation has encountered compliance and enforcement difficulties, at least in relation to the primary loot box mechanics in most (60.0–80.0%) top-grossing video games, probability disclosures are now provided, particularly in Asian jurisdictions (*e.g.*, Xiao & Lund, 2025; Xiao & Park, 2025; cf. Xiao, 2025). The same regulatory requirement mandating transparency should also apply to physical products that are similarly subject to consumer law.

Our study of 50 physical card packs purchased in Hong Kong revealed that the vast majority (80.0%) failed to provide *any* probability disclosures on their packaging, whilst the remaining 20.0% only provided limited and incomplete information as to the consumer's odds of winning rare prizes. The probability disclosures we examined revealed the high complexity of these product's rarity distribution, which in one example had to be presented by the manufacturer as a table spanning four separate pages on a PDF document (Topps, 2024). Faced with such overwhelming ‘information overload,’ consumers, particularly children, would struggle to fully comprehend the vast amount of information provided and the statistical implications (see Arnold, Goldschmitt, & Rigotti, 2023) and may even decide not to consult the probabilities at all, significantly reducing the efficacy of the intervention. In one extreme case, we found a disclosure stating that the highest rarity of cards could only be found in one out of 800,512 packs, which equates to a 0.00012492% chance, similar to winning one of the top prizes through the lottery (see Purkess, 2024). Indeed, a globally unique card that was manufactured only once from a recent *Magic: The Gathering* card pack reportedly sold for US$2 million (Kim, 2023). The consumer's probability of opening that specific lottery jackpot-like card from a card pack was disclosed by the manufacturer as ‘<0.00003%’ (Wizards of the Coast, 2023b).

Manufacturers ought to reconsider how they can meaningfully communicate the probabilities to consumers in an accessible manner: whilst we earlier criticised certain card packs for providing only limited probability information (and not the probabilities for obtaining every single card, as is required by consumer law), that lower level of detail might in fact be what many consumers would find most useful. Summarising the most important information (the probabilities of obtaining a card of various rarities and of the most desirable individual cards, which could be presented using only a handful of easily understandable percentage values) on the pack's physical packaging and then hyperlinking more curious consumers to the full details on a webpage would ensure both practical efficacy and regulatory compliance.

Possibly due to wider social and cultural acceptance as a result of having already been available on the market for many decades and multiple human generations, physical gambling-like products have not been subject to much regulatory scrutiny (Elliott & Mason, 2003). Regulatory standards, such as consumer law that governs all commercial products, should be evenly applied across industries. In principle, proponents of the video game industry were not wrong to highlight, in contrast to loot boxes, the wide acceptance of—and the lack of regulatory scrutiny against—physical gambling-like products (*e.g.*, Schreier, 2017; Valve Corporation, 2026), although scientific evidence suggesting that the physical products are less harmful may justify any divergent treatment (Xiao et al., 2026, p. 80). However, following that logic, instead of acceding to the video game industry demands that loot boxes should be scrutinised *less*, a more public health-oriented perspective would, on the contrary, argue that physical card packs should be scrutinised *more*.

Recent developments in card pack product design are making these products more gambling-like and financially risker than before, such as specifically designing card pack opening experiences to increase the excitement consumers feel (Rosewater, 2019), inserting extremely high-value, lottery jackpot-like prizes (Kim, 2023), and offering ‘collector’ editions of card packs with more exclusive content that are priced five times higher than normal packs, even though they contain the same number of cards (Rosewater, 2019). Limited product supply that cannot meet increasing demand has also meant that consumers often must resort to the secondary market to purchase card packs (Lucas, 2025; Muroi, 2026), whose value therefore fluctuates, thereby transforming the card packs themselves into risky speculative financial products.

Given card packs' notable resurgence in popularity following the Covid-19 pandemic (Ang, 2025; Igarashi, 2026; Lucas, 2025) and the emergence and popularisation of other novel forms of physical gambling-like products, such as blind boxes offering random toy figurines, *e.g.*, Labubus (Lee, Wyllie, & Brennan, 2025; Xiao, 2022), policymakers, regulators, and researchers should (re)assess these products as to their potential risks to public health. Mainland China has already required blind boxes (which was broadly defined and thus inclusive of physical card packs) to disclose probabilities (Xiao, 2022), and Singapore is expected to impose new regulations on physical gambling-like products, including card packs and blind boxes, by explicitly mandating standardised probability disclosures (Sun, 2026). Policy implementation in those jurisdictions should be specifically assessed. Our sample included only three Mainland Chinese packs in Simplified Chinese (6%), all of which (100%), encouragingly, did disclose probabilities (albeit without sufficient details) and state that they were unsuitable for young children aged under 8 (as is also required under the blind box regulations). In comparison, only seven of 47 non-Simplified Chinese packs (14.9%) disclosed probabilities, suggesting possible regional variations due to regulation. Other countries should consider adopting similar rules to guarantee transparency.

## Conclusion

Card packs are physical gambling-like products that offer random prizes in exchange for money that are deemed suitable for and are popular with children and adults alike. Unlike video game loot boxes, these physical products have faced little academic and regulatory scrutiny, despite posing financial harm and other potential addiction-related risks. We analysed 50 card packs purchased in Hong Kong and revealed that the vast majority (80.0%) failed to provide on their physical packaging any information as to the consumer's odds of winning rare prizes or hyperlinks to such information on webpages. The 20.0% of card packs that did provide probability disclosures failed to give sufficient details and were, in some cases, difficult to find and misleading. No pack (0.0%) allowed the consumer to know their odds of getting every individual card. In the absence of gambling law or dedicated regulations, consumer law should be better enforced in the context of physical gambling-like products to mandate transparency and enable consumers to make more informed purchasing decisions, emulating the limited but still recognisable progress already made in the digital video game loot box context. The potential behavioural addiction harms posed by novel gambling-like products should be further studied and properly regulated.

## Data Availability

Photographs recording the features of all physical card packs studied and screenshots of relevant webpages are publicly archived in the Open Science Framework at: https://doi.org/10.17605/OSF.IO/ZP7SX.
