# Correction to: Unpacking non-transparency: Most physical trading card game and collectible sports card packs failed to disclose probabilities for winning rare prizes

**DOI:** 10.1556/2006.2026.11197

**Published:** 2026-09-28

**Authors:** Ching Yiu Lo, Kuma Xiaoyu Xiong, Leon Y. Xiao

**Affiliations:** 1 School of Creative Media, City University of Hong Kong, China; 2 BeClaws.org, UK

Journal of Behavioral Addictions (2026) advanced online publication.

https://doi.org/10.1556/2006.2026.00197.

The authors identified an error in the Discussion section of the manuscript. Specifically, in the last two sentences of the second paragraph, the originally published text (with the erroneous text highlighted in bold italic):

Indeed, a globally unique card that was manufactured only once from a recent *Magic: The Gathering* card pack reportedly sold for US$2 million (Kim, 2023). The consumer's probability of opening that specific lottery jackpot-like card from a card pack was ***never*** disclosed by the manufacturer (Wizards of the Coast, 2023b).

The above paper has been modified as follows (with the incorrect word ‘never’ deleted and the correction highlighted in bold):

Indeed, a globally unique card that was manufactured only once from a recent *Magic: The Gathering* card pack reportedly sold for US$2 million (Kim, 2023). The consumer's probability of opening that specific lottery jackpot-like card from a card pack was disclosed by the manufacturer **as ‘<0.00003%’** (Wizards of the Coast, 2023b).

The original publication has been corrected upon the request of the corresponding author.

